# Supplementary material for: Particle-sounding of the spatial structure of kinetic Alfvén waves
Source: Nat Commun. 2023 Apr 12;14:2088. doi: 10.1038/s41467-023-37881-3 (PMC10097679; doi:10.1038/s41467-023-37881-3)
Supplement: Supplementary file 1 — Supplementary Information [file 41467_2023_37881_MOESM1_ESM.pdf]

Supplementary Information for

Particle-sounding of the Spatial Structure of Kinetic Alfvén Waves

Z.-Y. Liu, Q.-G. Zong*, R. Rankin, H. Zhang, Y.-X. Hao, J.-S. He, S.-Y. Fu, H.-H. Wu, C. Yue, C. J. Pollock, G. Le

*Correspondence to: qgzong@pku.edu.cn

This PDF file includes:

Supplementary Figures 1 to 4
Supplementary Table 1

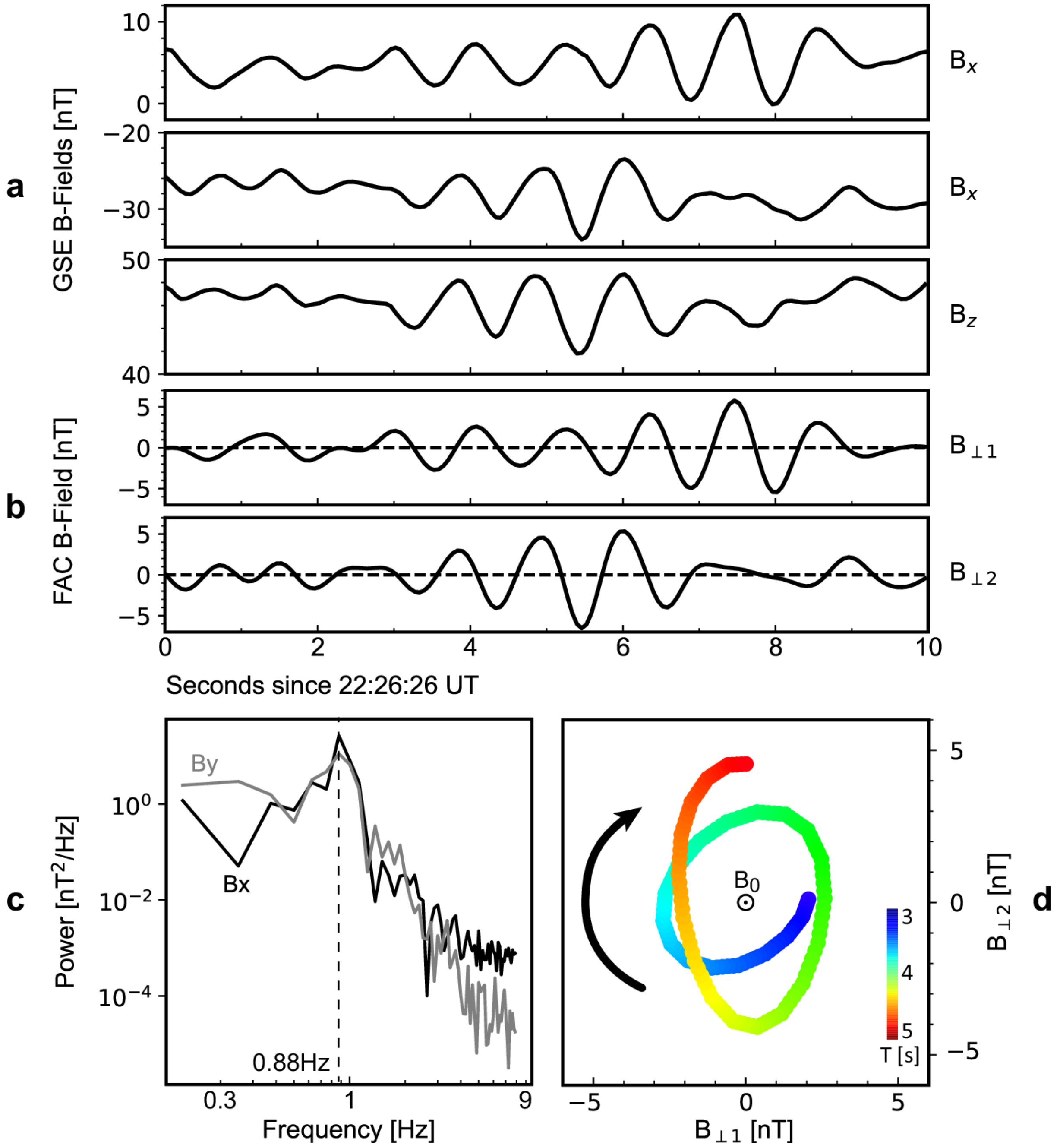

Supplementary Fig. 1. The magnetic fields of the KAWs. (**a**) The magnetic fields in the GSE coordinates. (**b**) The magnetic fields in the FAC system. (**c**) The Fourier-transform power of the magnetic fields. (**d**) The $B_{\perp 1}$-$B_{\perp 2}$ hodogram.

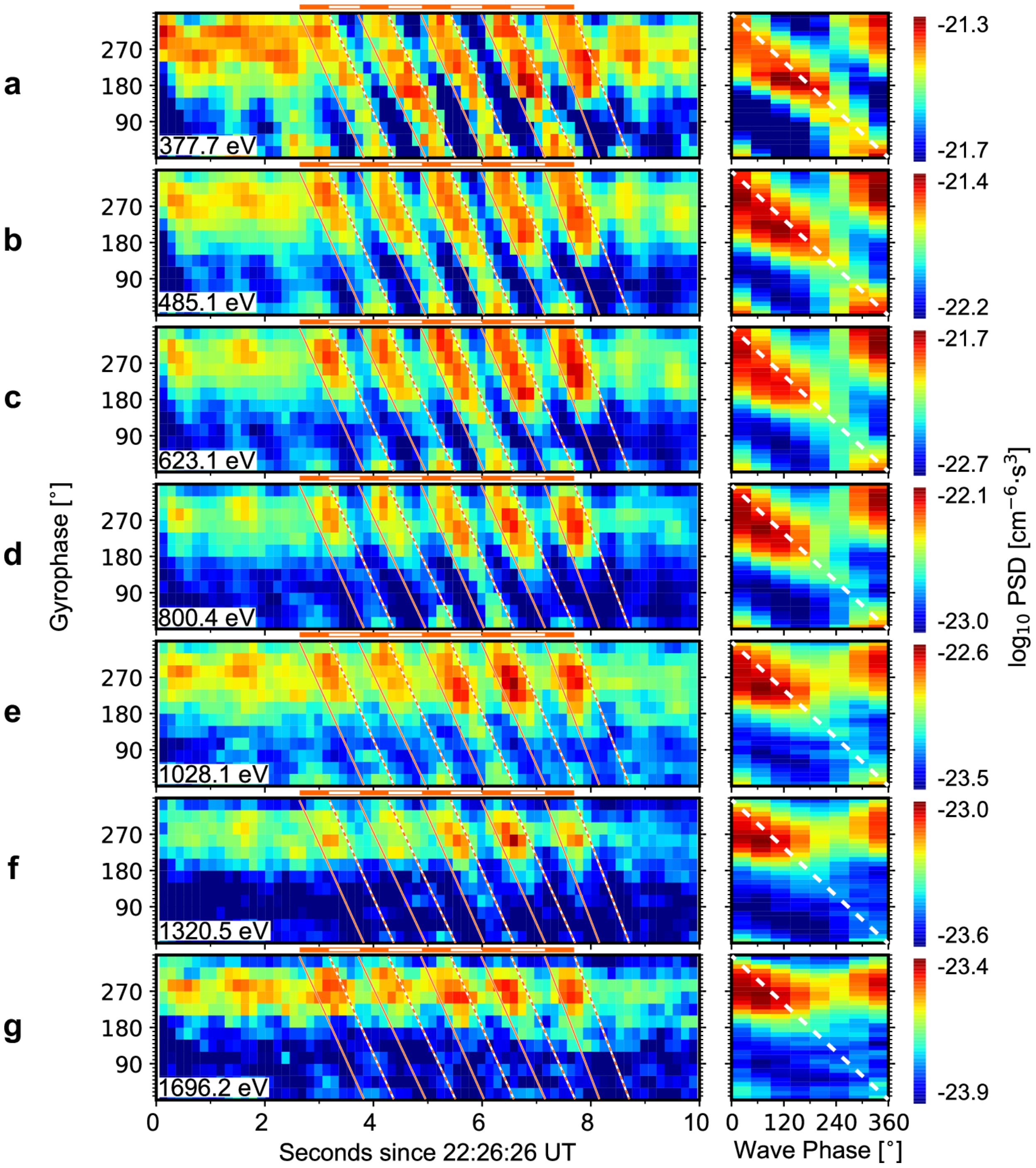

Supplementary Fig. 2. The gyrophase-time spectrograms and gyrophase-wave phase spectrograms of proton PSDs. (a-d) correspond to different energy channels of the FPI-DIS instruments. The solid and dashed lines in all panels give the expected positions of phase-bunching stripes.

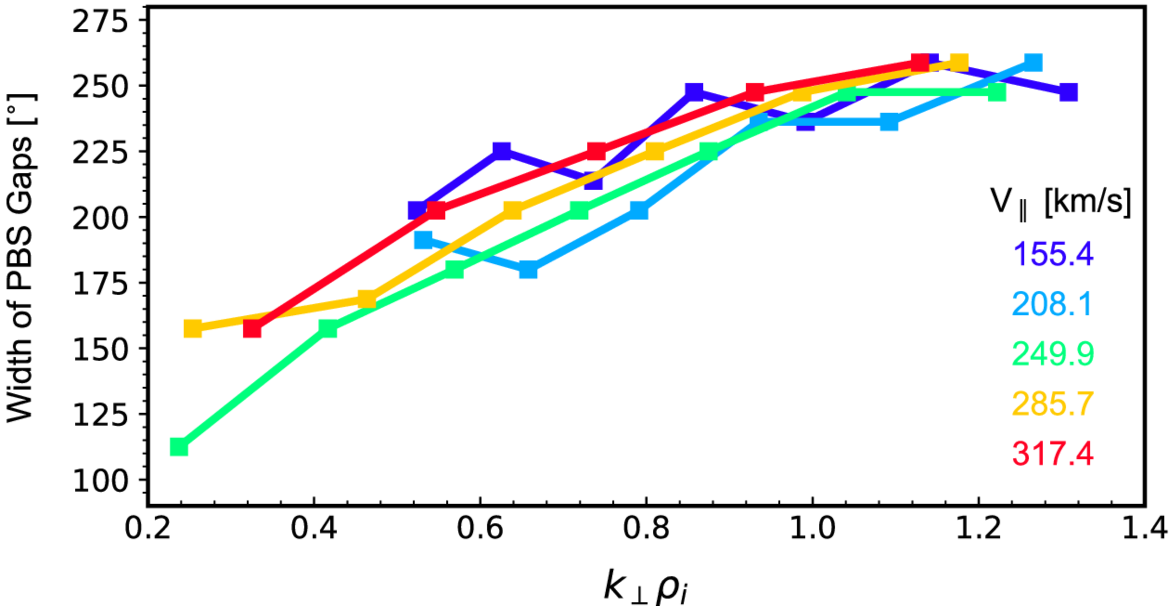

Supplementary Fig. 3. Gyrophase extent of phase-bunching stripe gaps, defined according to the 0.8-level curve shown in Fig. 4b. Different colors correspond to different parallel velocities.

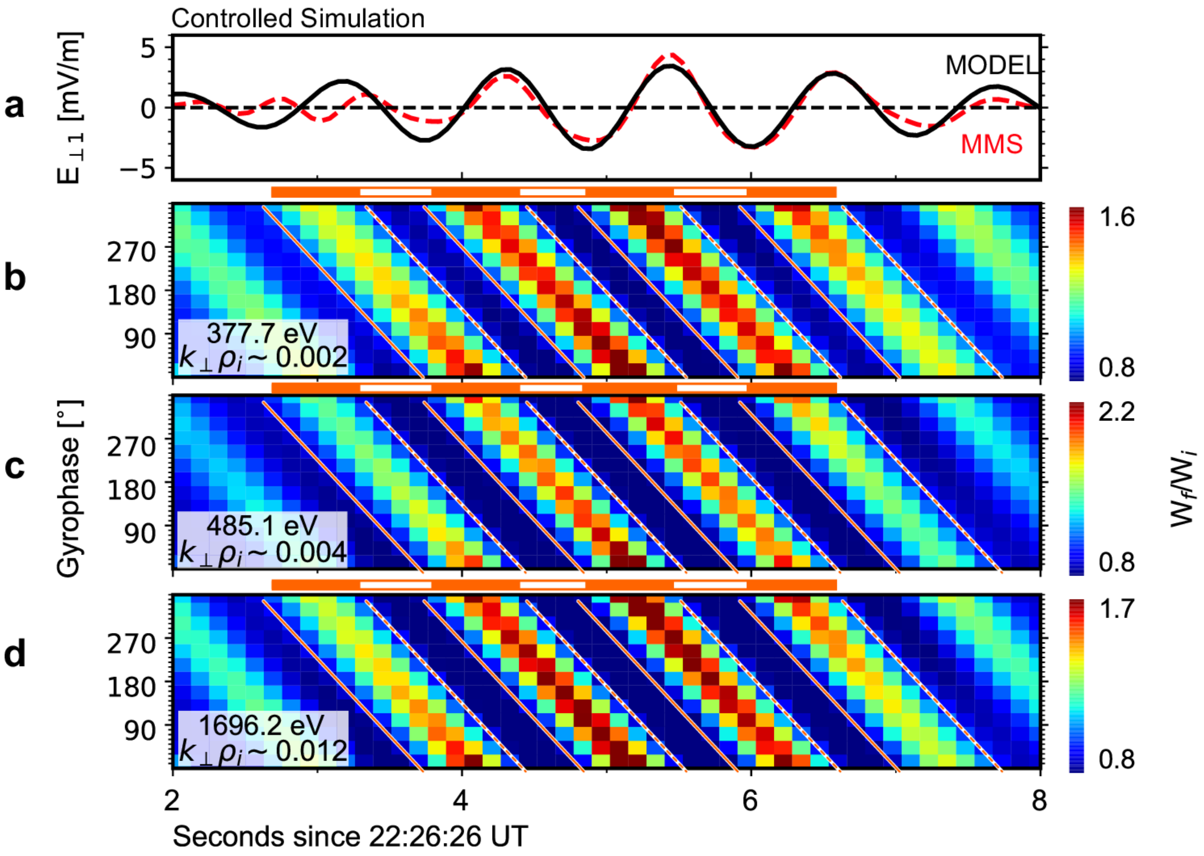

Supplementary Fig. 4. The controlled simulations. (**a**) The modeled KAW electric fields (the black curve) and the observations (the red curve). (**b-d**) The ratio between the final and initial proton energy. The orange solid and dashed lines give the expected positions of phase-bunching stripes. No phase-bunching stripe gaps are observed.

Supplementary Table 1. KAW events shown in Fig. 6. In this table, E_u represents the highest energy channel in which gyrophase-bunching stripes can be identified, PA denotes the pitch angle at which gyrophase-bunching stripes are clearest, B represents the magnitude of the background magnetic field, $T_\perp$ represents the perpendicular temperature of protons taken from the MMS/FPI/DIS-MOMS dataset, $\lambda_\perp$ represents the perpendicular wavelength of KAWs given by the particle-sounding technique, $\rho_{i,th}$ gives proton thermal gyro-radius, R denotes MMS spacecraft separation, $B_\perp$ and $E_\parallel$ represent the amplitude of wave perpendicular magnetic fields and parallel electric fields, and WNA stands for wave normal angle. The first event is the one analyzed in the main text.

Date	UT	E_u [eV]	PA [∘]	B [nT]	$T_\perp$ [eV]	$\lambda_\perp$ [km]	$\rho_{i,th}$ [km]	$\lambda_\perp$ $/R$	$B_\perp$ [nT]	$E_\parallel$ [mV/m]	WNA [∘]
2015/12/30	22:25:29-22:26:34	1696	112.5	55.1	340	99.8	48.4	2.3	3.7	0.67	88.5
2016/01/27	07:15:34-07:15:39	800	112.5	62.2	218	60.7	34.3	3.6	3.4	0.55	87.4
2016/02/15	01:03:48-01:03:53	1696	135	43.9	216	95.9	48.4	6.7	2.2	0.42	89.1
2016/10/13	12:48:20-12:48:28	3967	90	44.0	1676	206.8	134.4	27.9	1.0	0.47	89.8
2016/10/22	10:28:47-10:28:57	1696	90	54.8	270	108.7	43.3	10.7	2.1	0.42	83.4
2016/12/05	05:16:28-05:16:34	2263	78.75	50.0	269	134.9	47.4	18.7	1.9	0.38	82.0
2016/12/05	05:20:58-05:21:06	736	90	57.4	259	68.3	40.5	9.4	3.8	0.48	86.5
2016/12/30	14:08:51-14:08:57	1291	90	56.8	327	91.4	46.0	11.1	1.7	0.42	85.8
2016/12/31	02:37:19-02:37:25	556	90	61.1	266	55.8	38.6	4.9	1.3	0.24	89.9
2017/01/11	11:22:50-11:22:58	2263	90	48.4	536	141.9	69.1	17.7	1.2	0.33	77.2
2017/01/12	11:44:18-11:44:24	3967	123.75	48.5	386	155.9	58.5	23.6	4.3	0.97	86.0
2017/01/24	23:58:43-23:58:53	2263	90	58	298	118.4	43.0	13.5	2.2	0.24	81.5
2018/01/07	02:41:31-02:41:41	1696	90	54.3	179	109.6	35.6	3.9	1.1	0.46	83.3
2018/12/08	06:48:40-06:48:47	4752	90	46	547	216.7	73.5	7.6	4.4	0.84	89.8
2020/04/21	12:08:06-12:08:12	2802	56.25	50.6	131	125.8	32.7	2.1	5.1	0.98	89.7
